# Developing medical education capacity in Russia: twenty years of experience

**DOI:** 10.1186/s12909-017-0861-z

**Published:** 2017-01-25

**Authors:** Bulat A. Ziganshin, Mitra Sadigh, Liliya M. Yausheva, Anna P. Ziganshina, Arseniy A. Pichugin, Alexey S. Sozinov, Nail Kh. Amirov, Asghar Rastegar, Ayrat U. Ziganshin, Majid Sadigh

**Affiliations:** 1 Kazan State Medical University, Kazan, Russia; 2 Yale University School of Medicine, New Haven, CT USA; 3 Western Connecticut Health Network, 24 Hospital Ave, Danbury, CT 06810 USA; 4 University of Vermont Robert Larner, M.D. College of Medicine, Burlington, VT USA

**Keywords:** Clinical medical education, Capacity building, International partnership, Long-term results

## Abstract

**Background:**

The partnership between Yale University (USA) and Kazan State Medical University (KSMU, Russia) was established in 1996 and transitioned to Western Connecticut Health Network (WCHN)/University of Vermont Robert Larner, M.D. College of Medicine (USA) in 2012 with the goal of modernizing medical education at KSMU primarily through introduction of the American medical education structure, role modeling, and educational capacity building. It was centered on the formation of a select group of Russian junior faculty members familiar with American medical education who would then initiate a gradual change in medical education at KSMU. Here we describe the 20 year partnership, rooted in local capacity building, through which a sustainable, mutually rewarding international collaboration was established. In addition, we evaluate the program’s outcomes and impact on medical education at Kazan State Medical University, and assess its influence on Russian program participants.

**Methods:**

Senior residents and faculty were sent to KSMU to conduct teaching sessions with local faculty and trainees. Their responsibilities included familiarizing Russian colleagues with specific topics in clinical medicine, importing knowledge about the basics of teaching, clinical epidemiology and evidence based medicine, and creating, in consistency with the American model, a “Clinical Teaching Team Structure” that integrates patient care with clinical education. Furthermore, 44 of selected KSMU members, including 13 junior faculty (29.5%), 14 clinical PhD students (31.8%), 12 interns/residents (27.3%), and five medical students (11.4%), were trained at Yale/WCHN or one of their major affiliated community hospitals for a period of 1 to 12 months for a total of 844 participant-weeks of training.

**Results:**

Thirty (68.2%) individuals who were trained in the U.S. are currently working in Kazan primarily as faculty at KSMU. Among them, three trainees (10%) have become heads of their department, eight (26.7%) hold senior faculty positions, and two (6.7%) have clinical and educational administrative leadership positions. Two major clinical departments have adopted the “Clinical Teaching Team Structure.” As a result of the collaboration, three teaching courses – Evidence-Based Medicine, Tropical Medicine, and HIV/AIDS Medicine – have been designed and incorporated into the curriculum.

**Conclusion:**

This partnership has been instrumental in introducing the American medical education model and expanding the medical knowledge of faculty, residents, and students of KSMU on infectious diseases, HIV/AIDS, tropical medicine, renal diseases, and global health topics. Capacity building through the Yale/WCHN-KSMU exchange program has greatly contributed to the quality of medical education at Kazan State Medical University.

**Electronic supplementary material:**

The online version of this article (doi:10.1186/s12909-017-0861-z) contains supplementary material, which is available to authorized users.

## Background

At the end of the 20^th^ century, the healthcare system in post-Soviet Russia was in a state of steady decline [1] as the nation experienced poor life expectancy [2, 3], decreased birth-to-death ratio [4, 5], and an increase in cases of preventable diseases among the working adult population [6, 7]. This state of affairs was partially ascribable to the financial burdens of the centralized Soviet healthcare model, which was fiscally unsustainable in post-Soviet Russia. The quality of undergraduate and postgraduate medical education declined as higher medical educational institutions experienced similar funding shortages (see Additional file 1 for details regarding the system of medical education in Russia) [8]. Poorly equipped with antiquated resources, universities relied heavily on Soviet-era textbooks as the primary source of information. A lack of research funding coupled with the low prioritization of research activities in educational academic facilities resulted in a dearth of high-quality research projects published by Russian physicians/scientists in international mainstream journals. Similar trends were observed in other former Soviet Union countries [9].

In response to the deteriorating state of healthcare and medical education, the United States Agency for International Development (USAID) funded a 5 year grant project initiated in 1992 with the goal of modernizing the medical education system in Russia, Ukraine, and Belarus. This project involved 13 higher medical educational institutions in the former Soviet Union countries and several partnering institutions from the US. Kazan State Medical University (KSMU), located in Kazan, the capital city of the Republic of Tatarstan, was one of several participating institutions from Russia (please see Table 1 for key facts regarding KSMU) assigned to collaborate with the University of Rochester and Yale University School of Medicine (YSM). This collaboration made it possible for several faculty members from both American institutions to visit KSMU for 2 to 4 weeks to introduce and disseminate current medical information to medical students, residents, and faculty, while KSMU administrators and faculty visited the American institutions to obtain familiarity with the healthcare and medical education system in the United States.

**Table 1:** Key facts about Kazan State Medical University (Kazan, Russia)

Kazan State Medical University:
• Established in 1814, third oldest higher medical institution in Russia (after Moscow and St. Petersburg)
• 5500 students in total (undergraduate and postgraduate)
• 18.6% of students are international from over 54 countries
• 700 teaching faculty members
• Currently, KSMU functions as a college of health sciences and includes the following nine faculties (schools): General Medicine, Pediatrics, Preventive Medicine, Dentistry, Pharmacy, Social Work, Management and Higher Nursing Education, Medical Biochemistry, and Medical Biophysics
• Duration of study at the General Medicine Faculty is 6 years:
- 2.5 pre-clinical years
- 3.5 years of clinical studies

This initial USAID-sponsored project provided the platform for an important pilot phase that ultimately led to the establishment of an extended collaboration between YSM and KSMU by aiding the development of professional contacts between the leadership and key faculty of the two institutions. By 1996 when the USAID grant program was completed, a strong bilateral desire to continue the collaboration had formed with the aim of advancing undergraduate and postgraduate medical education in Kazan, primarily through faculty efforts in the Department of Medicine at YSM. In 2012 the collaboration transitioned to the Western Connecticut Health Network/University of Vermont Robert Larner, M.D. College of Medicine (WCHN/UVMCOM) Global Health Program. This newly formed partnership emphasized the value of trained faculty members familiar with modern medical education in the U.S. in initiating gradual change in medical education at Kazan State Medical University.

Therefore, on the 20^th^ anniversary of the establishment of this international collaboration, we describe our experience in establishing a productive and sustainable international collaboration rooted in local capacity building, evaluate its outcomes and impact on medical education at Kazan State Medical University, and assess its influence on Russian program participants.

## Methods

### Main goals of the collaborative program

The collaborative program between the Department of Medicine of YSM and KSMU was designed with the intention of improving the quality of medical education at KSMU. After several needs assessment meetings between the leadership and faculty of the two institutions, the following specific objectives were formulated:

- To introduce and establish the “Clinical Teaching Team Structure” in patient care, similar to that in the U.S. which includes one attending physician, one resident, one intern, and 1–2 medical students, by integrating undergraduate and postgraduate medical education with patient care. This system was completely novel to the Russian medical education and healthcare system.
- To integrate evidence-based medicine into the medical education curriculum.
- To familiarize Russian physicians with the American medical education and healthcare system.
- To familiarize American participants with the Russian healthcare system.
- To share and exchange Russian, Tatar, and American cultures and traditions.

### Description of the starting model

The collaborative program between YSM/WCHN/UVMCOM and KSMU included the following main items:

- **Establishment of a Medical Library (1996).** The collaboration began with the establishment of a medical library with English language resources and reliable Internet access. YSM donated medical textbooks, several computers, and paid subscriptions for electronic resources such as UpToDate (CD-disk version) in an effort to modernize KSMU’s library. These donations provided physicians, educators, and trainees (including medical students and residents) access to evidence-based information for teaching, research, and learning purposes. KSMU’s medical library has grown considerably over the years. It currently houses two full computer classrooms, an accessible and digitalized library catalogue, online evidence-based resources, and essential databases including Physician’s Consultant, Scopus, eLibrary, ClinicalKey, EBSCO Host, and Elsevier’s ScienceDirect. Although these accomplishments may seem minor, they set the foundation for all subsequent improvements.
- **English Language Teacher Training (1996).** The overwhelming majority of KSMU students, residents, and physicians lacked English language proficiency despite the inclusion of a medical English course in the university curriculum. Since utilization of the educational resources provided by YSM required working knowledge of English, a faculty member of KSMU’s Department of Foreign Languages was invited to the U.S. to attend a ten-week-long training program at the Yale English Language Institute to complete training in novel English language teaching methods.
- **Establishment of an International Office at KSMU (1996).** A dedicated office with English-proficient staff was needed at KSMU to coordinate collaborative efforts and to manage administrative tasks related to incoming and outgoing program participants. Hence, an office headed by an experienced KSMU faculty member with vast international experience, aided by two full-time staff members, was established. In 2009, the International Office was restructured and reformed into several departments, among them the Office of Global Health.
- **Transition of the Program from YSM to WCHN/UVMCOM.** In 2012, a key faculty member from the YSM Department of Medicine moved to WCHN/UVMCOM to establish a regional center for Global Health education [10], and the collaboration with KSMU transitioned in tandem. Consequently, all training from 2012 onward has been conducted at WCHN/UVMCOM.

### Clinical teaching in Russia and training in the US

#### Teaching at KSMU (1996–2016)

Every year, a group of YSM/WCHN/UVMCOM faculty members was selected to travel to Kazan to conduct comprehensive teaching seminars. The American group included twenty faculty members. In addition, ten senior residents from YSM travelled to Kazan for 1 to 2 month visits with three main goals: (1) to learn about the Russian healthcare and medical education system via direct participation in in-hospital and outpatient activities; (2) to teach junior residents and students of KSMU various medical topics; and (3) to exchange cultures, traditions, and ideas in healthcare and medical education.

During the annual visits to Kazan, YSM/WCHN/UVMCOM faculty prepared a robust teaching schedule for KSMU trainees and faculty. Teaching was conducted through lectures, seminars, ward rounds, and inpatient and outpatient consults. Table 2 lists the formal lecture courses that were frequently given over the years.

**Table 2:** List of lecture courses

Title of Lecture Course
● Evidence-based medicine (basic and advanced)
● General internal medicine topics (depending on the specialty of visiting faculty)
● Teaching how to teach (the Stanford Model)
● Nephrology (workshops and seminars)
● Annual HIV/AIDS medicine course
● Annual infectious diseases course
● Annual biostatistics and clinical epidemiology (basic and advanced) course
● Annual tropical medicine course
● Annual global health course

Teaching sessions were either directed to an English-proficient audience, or conducted with simultaneous translation. The audience for each training course was determined in advance and included senior residents, clinical PhD students,^1^ and junior and senior faculty members from KSMU. Although some courses such as tropical medicine were specifically intended for medical students, all courses offered open attendance to ensure that anyone could attend irrespective of the training level. All participants received a certificate of completion at the end of the course.

Collaborative efforts were extended beyond Kazan to neighboring Naberzhnye Chelny, the second largest city in the Republic of Tatarstan, home to KSMU’s distant campus. Visits to Naberzhnye Chelny were shorter in duration, usually spanning 5 to 7 days, and focused on general medical topics including HIV/AIDS.

#### Training KSMU faculty and trainees at YSM/WCHN (1996–2016)

Russian program participants willing to travel to the U.S. were competitively selected to undergo training in Connecticut in various hospitals affiliated with YSM and WCHN/UVMCOM. Selection of participants was based on the following four criteria: (1) Excellent track record at KSMU (as a trainee and/or employee) as evidenced by grades/supervisor recommendations; (2) Knowledge of English language; (3) Recommendation of the Rector and/or Vice-Rector of KSMU; and (4) Motivation to institute change in medical education at KSMU upon return to Russia.^2^ Following a thorough application process participants were selected after in-person interviews of all applicants by a YSM/WCHN/UVMCOM faculty member.

The hosting institution developed the curriculum for training Russian program participants in the US. The following teaching hospitals in Connecticut were involved in this training: Yale-New Haven Hospital (New Haven), Waterbury Hospital (Waterbury), West Haven VA Hospital (West Haven), St. Mary's Hospital (Waterbury), and Danbury Hospital (Danbury).

The goal of the training program was to familiarize participants with the systems of graduate and postgraduate medical education and patient-centered healthcare in the US. To this end, all program participants were assigned to a general medical team for a minimum of 4 weeks, regardless of their specialty. This assignment allowed maximal exposure to a wide variety of diseases and educational opportunities. Program participants spent the remaining time on their respective specialties with special emphasis on evidence-based decision-making in patient care. All participants were actively integrated into medical teaching teams. They were also expected to attend daily morning reports and noon conferences, as well as all teaching sessions organized by the hosting hospitals/departments including Grand Rounds, continuous medical education (CME) seminars, and global health events, and to participate in multiple medical educational committees including the house staff evaluation and ethical committees.

Over the course of the 20 year collaboration, 44 Russians trained in the US, all of whom were affiliated with KSMU either as employees or trainees at the time of enrollment (see Table 3 for detailed characteristics of participants). Among the participants, 42 (95.5%) had some degree of clinical training while one (2.3%) was an English language teacher and one (2.3%) a basic scientist.

**Table 3:** Profile of participants of the Yale School of Medicine/Western Connecticut Health Network—Kazan State Medical University exchange program

Variable	Value	Percentage (%)
Total Number of Participants	44	100%
Mean Age ± Standard Deviation	27.8 ± 6.2	—
Age Range	19–49	—
Males	22	50.0%
Females	22	50.0%
Level of Medical Education at the time of participation:		
Medical Student	5	11.4%
Clinical Resident	12	27.3%
Clinical PhD Student^a^	14	31.8%
Junior Faculty (Lecturer, Assistant Professor, Associate Professor)	13	29.5%
Duration of Training in the US:		
1 months	2	4.5%
2 months	6	13.6%
3 months	7	15.9%
4 months	13	29.5%
5 months	3	6.8%
6 months	10	22.7%
8 months	1	2.3%
12 months	2	4.5%

^a^In Russian postgraduate medical education, a “clinical PhD student” is an analogue of a Fellow in the U.S.-based medical education

Participants who underwent clinical training (*n* = 42) at YSM/WCHN/UVMCOM-affiliated hospitals averaged 4 month durations (range 1 to 12 months), comprising a total of 783 participant-weeks of training. All 42 participants (100%) rotated through the Internal Medicine service for a minimum of 4 weeks, while medical students and those specializing in Internal Medicine in Russia spent the entire duration of the exchange program on this service. Participants not specializing in Internal Medicine rotated through other departments, including infectious diseases (*n* = 8), obstetrics and gynecology (*n* = 5), neurology (*n* = 3), ophthalmology (*n* = 3), cardiology/cardiac surgery (*n* = 2), gastroenterology, nephrology, neurosurgery, general surgery, oncology, rheumatology (*n* = 1 each). One participant underwent specialized training in EBM and one in the Stanford Model for Faculty Development.

### Evaluation methods – surveying program participants

All Russian participants of the KSMU/YSM-WCHN/UVMCOM exchange program who underwent training in the U.S. were asked to complete a general post-rotation questionnaire (see Additional file 2 for the survey tool). Of the 44 exchange program participants, 43 (97.7%) completed and returned the questionnaire, while one participant was unable to be reached. A subgroup of the program participants (*n* = 15) who are currently employed at KSMU were surveyed using a post-rotation questionnaire that was developed in conjunction with YSM (see Additional file 3 for the survey tool) aimed at assessing the training at Yale/WCHN-UVMCOM as well as the current and past attitudes in clinical medical education. Since this questionnaire was targeted mainly at clinicians, two participants who were not trained in clinical medicine, an English teacher and a basic scientist, were excluded from this analysis.

### Funding

The first 5 years of the program were funded by a grant from USAID. Following this grant, funding was provided by donating organizations including the Federation of Jewish Communities of Western Connecticut, Nephrology and Hypertension Associates, Saint Mary’s Hospital, Waterbury Medical Association, Yale Department of Global Health Office, Yale Johnson & Johnson Physician Scholars Program, WCHN Global Health Program, and KSMU.

## Results

The main results of the long-standing collaboration amongst KSMU and YSM/WCHN/UVMCOM can be classified into two main categories: (1) training of Russian physicians in the U.S.; and (2) changes to KSMU’s curriculum. The training of Russian physicians and medical trainees in the U.S. was a major component of capacity building and improvement of medical education and healthcare in Kazan. Each program participant received personalized training based on individual preferences and goals. In this section we provide the results of the evaluation of Russian physicians who trained in the U.S. and describe the consequential changes to the clinical teaching methods at KSMU.

### Results of the evaluation

#### Views on exchange program

Forty-three (97.7%) of the 44 participants completed the post-rotation questionnaire. They indicated improvement of clinical knowledge and skills (*n* = 31, 72.1%), familiarization with the American system of medical education and healthcare (*n* = 21, 48.8%), conduction of research (*n* = 7, 16.3%), and improvement of English language skills (*n* = 5, 11.6%) as their main aims of participation in the exchange program.^3^ One participant (2.3%) indicated mastering the American system of medical education and evidence-based medicine, and subsequent adoption of these principles for KSMU-trainee medical education, as the main aim. Throughout their time in the U.S., participants took part in daily clinical and educational activities. Some were also involved in research projects (for details see Table 4). Overall, participants were extremely satisfied with their training experience in the U.S., with ten participants indicating that the program met 75% of their expectations, while it met 100% of expectations for 33 participants, averaging 94% of expectations met overall. Participants expressed high satisfaction with the exchange program’s level of organization (Table 5).

**Table 4:** Participation in daily activities throughout the exchange program

Variable	Value	Percentage (%)
Number of participants undergoing clinical training^a^	41	100%
Main Clinical Activities:		
Observing/assisting in the operating room	9	22.0%
Attending patients on the wards	35	85.4%
Working in outpatient clinics	32	78.0%
Daily rounds with the attending physician and team	41	100%
Overnight shifts in the hospital	25	61.0%
Educational Activities:		
Daily morning conferences	41	100%
Daily noon conferences	41	100%
Grand Rounds (weekly)	40	97.6%
Working with medical students	22	53.7%
Basic science or clinical research activities	9	22.0%
Usage of literature resources (library, online databases):		
Every day	41	100%
Once a week	0	0%
Less than once a week	0	0%

^a^Excludes two non-clinical participants of the program (English language teacher and basic scientist) and one clinical participant who did not complete the post-rotation survey

**Table 5:** Evaluation of satisfaction by various aspects of the exchange program by participants (the following scaling system was used: 1 – Very Poor, 2 – Poor, 3 – Satisfactory, 4 – Good, 5 – Excellent)

Variable	Value ± SD
Number of participants^a^	43 (100%)
Score averages across participants:	
Exchange program organization in general	4.79 ± 0.47
The role of the hosting organizers (US)	4.91 ± 0.29
The role of the local organizers (Russian)	4.70 ± 0.64
Boarding	4.53 ± 0.83
Accommodation	4.73 ± 0.60
Travel arrangements	4.81 ± 0.50
Financial support	4.44 ± 1.01
Availability of main resources	4.84 ± 0.37
Hosting personnel	4.95 ± 0.21

^a^Excludes one clinical participant who did not complete the post-rotation survey

#### Current employment status of participants

**Fig. 1:**
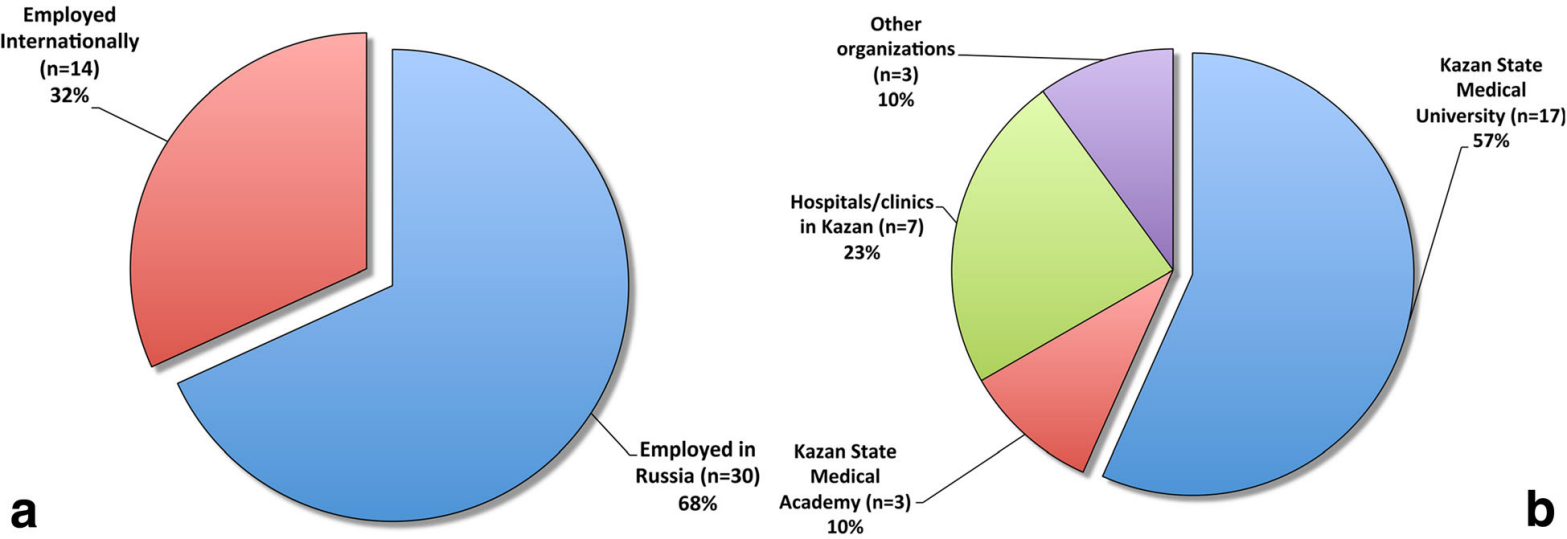
Employment status of exchange program participants: Panel **a** – shows a comparison of employment in Russia and abroad; Panel **b** – illustrates in detail the institutions, at which the participants are employed within Russia

Upon evaluation of program participants’ current employment status (Fig. 1), it was found that two-thirds are employed within Russia, and 57% (*n* = 17) of that group are employed at KSMU. Three participants (10%) are employed at Kazan State Medical Academy.^4^

#### Survey results of KSMU employees

**Fig. 2:**
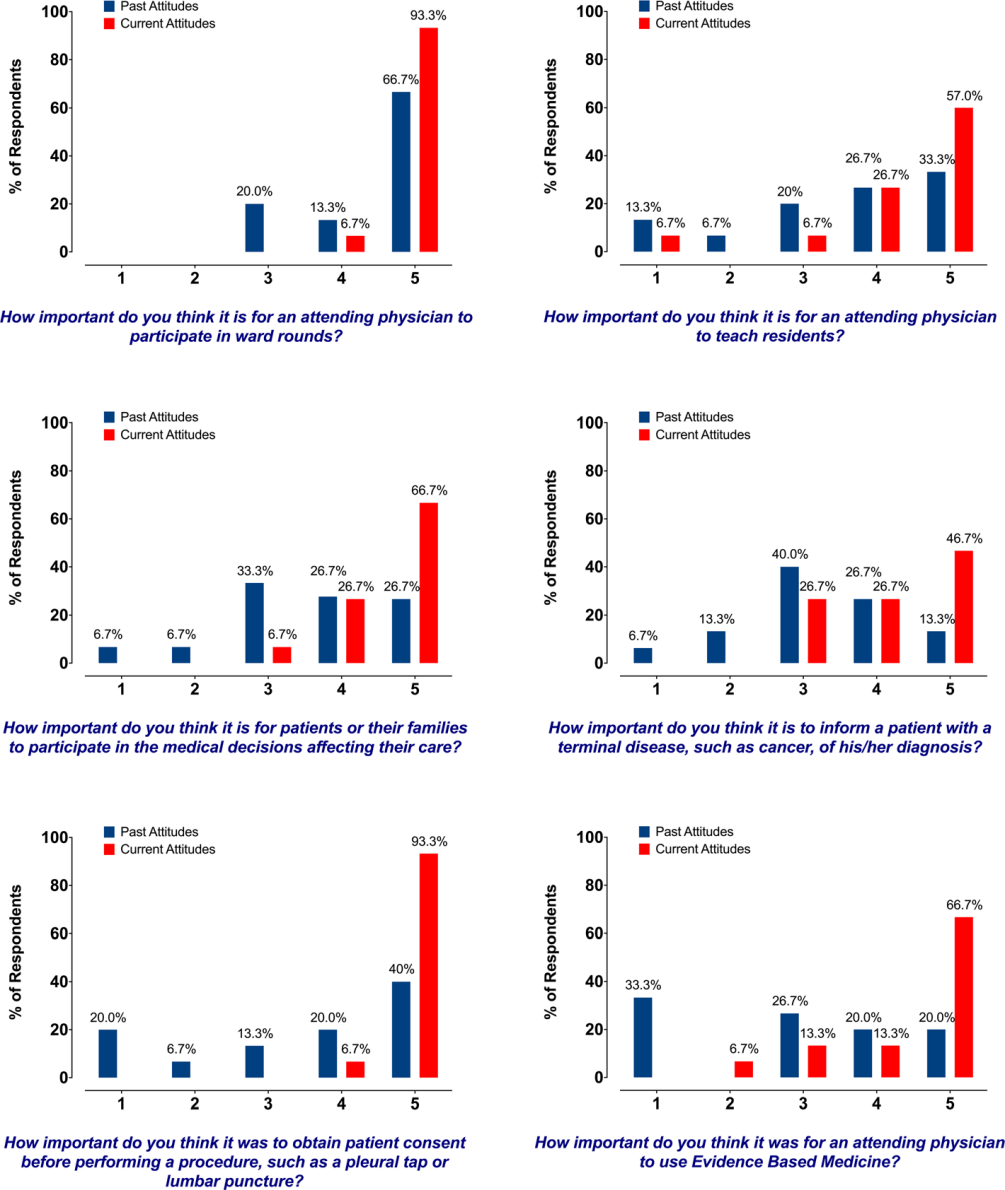
Selected survey results of exchange program participants who are currently employed at Kazan State Medical University (*n* = 15) illustrating the past and current attitudes towards various aspects of clinical education. Scaling system: 1 – not important at all; 5 – very important. (Additional survey results are available in Fig. 1a in Additional file 4)

**Fig. 3:**
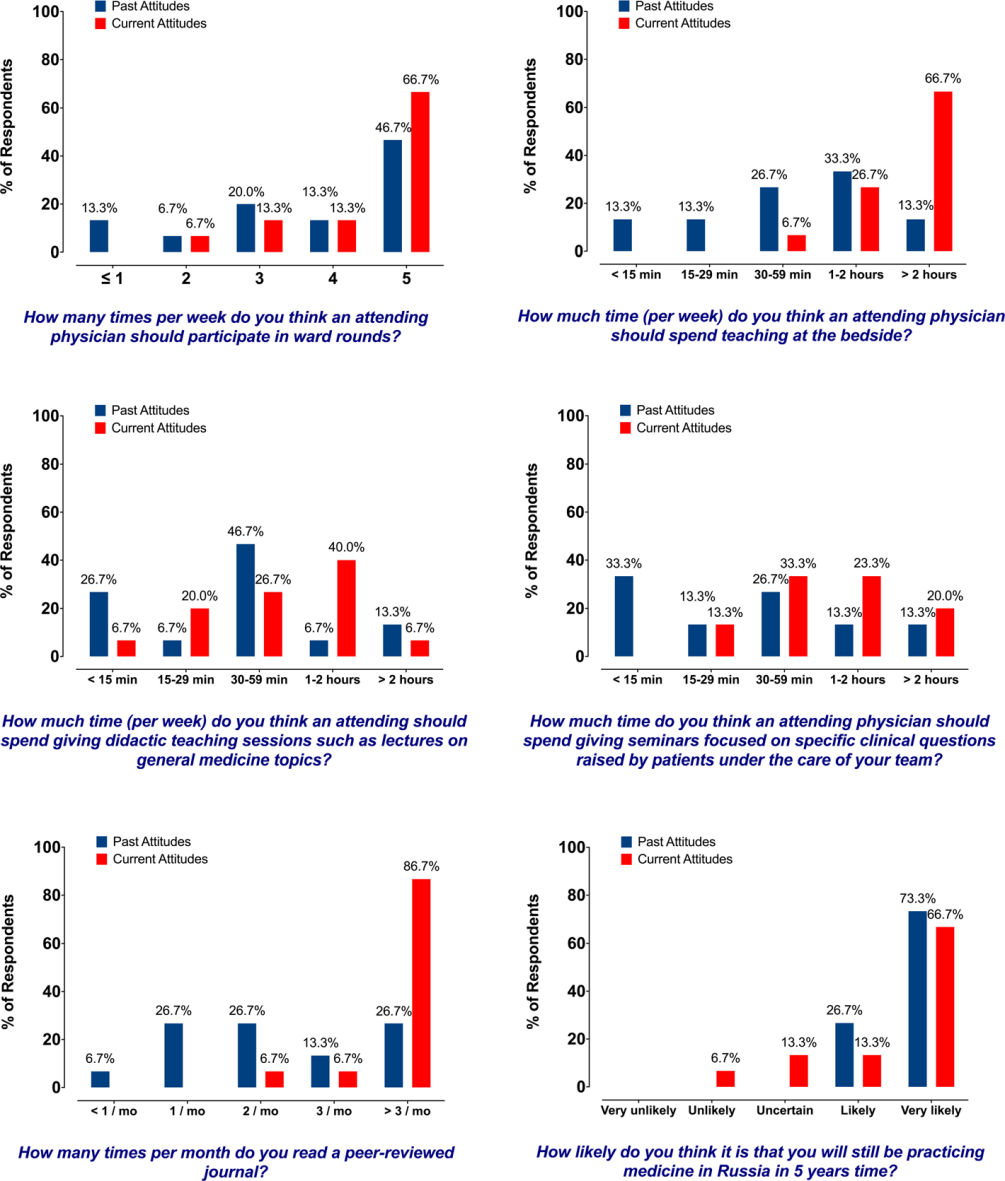
Survey results of exchange program participants who are currently employed at Kazan State Medical University (*n* = 15) illustrating the past and current attitudes towards various aspects of clinical education

A subgroup of exchange program participants (*n* = 15, excluding the English-language teacher and the basic scientist), currently employed at KSMU, completed a separate more detailed questionnaire regarding clinical education (Additional file 3). It is clear from comparing KSMU participants’ past and current attitudes that the rotation had a very strong impact on participants (Fig. 1a in Additional file 4, Fig. 2 and Fig. 3). A shift towards an increased importance is noted in all observed responses across all questions, signifying Russian program participants’ adoption of the U.S. model of education. The most striking differences between current and past attitudes are observed in important clinical issues such as obtaining consent prior to a procedure and involvement of family participation in the decision making process (Fig. 1a in Additional file 4 and Fig. 2). Additionally, participants are keen to acknowledge the importance of evidence-based medicine in daily clinical practice, an attitude that is markedly different from the pre-rotation responses (Fig. 1a in Additional file 4 and Fig. 2). Participants also learned the importance of reading articles from peer-reviewed journals on general medical topics and studies pertaining to their own specialty (Fig. 3). Finally, fewer KSMU employees were certain of their professional future in Russia after completing training in the U.S. (Fig. 3).

**Fig. 4:**
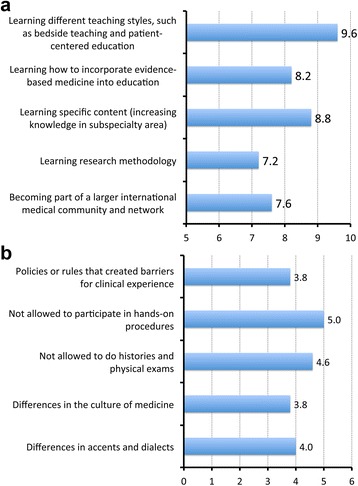
Potential benefits (Panel **a**) and challenges (Panel **b**) of participating in a clinical rotation in the US: rated in accordance of importance to the participants on a 1 to 10 scale (1 – not important at all, 10 – very important)

Upon more detailed evaluation of their training in the U.S., participants ranked the potential benefits and challenges of the rotation in order of importance (Fig. 4). Learning different teaching styles, increasing knowledge of skills in a certain area, and incorporating evidence-based medicine into education were ranked as the top three benefits (Fig. 4, panel a), while the main challenges were related to regulatory legal policies in the U.S. medical educational hospitals that prohibited hands-on experience (Fig. 4, panel b). Twelve participants (80%) characterized their Yale/WCHN/UVMCOM mentors as very good, and the remaining three participants (20%) as good. More than half (60%) of the KSMU participants confirmed that they are still academically involved with their mentors or involved in projects with Yale/WCHN/UVMCOM affiliated colleagues. As a result of training in the US, participants noted a gain in specific clinical (93%, *n* = 14), teaching (80%, *n* = 12), and research skills (60%, *n* = 9).

Upon return to Russia, seven participants (47%) acknowledged having encountered certain challenges including misunderstandings in the workplace, acquisition of unneeded skills at Yale/WCHN/UVMCOM, culture shock, lack of understanding of evidence-based medicine among Russians colleagues, disappointment from an inability to implement obtained skills upon return, and unpreparedness of the Russian medical society to make changes. Fourteen participants (32%) elected to continue their careers abroad. Eleven (78.6%) of participants in this group were either a medical student, resident, or PhD student at the time of their participation in the program, and five (35.7%) were female.

#### Career development of KSMU exchange program participants

**Fig. 5:**
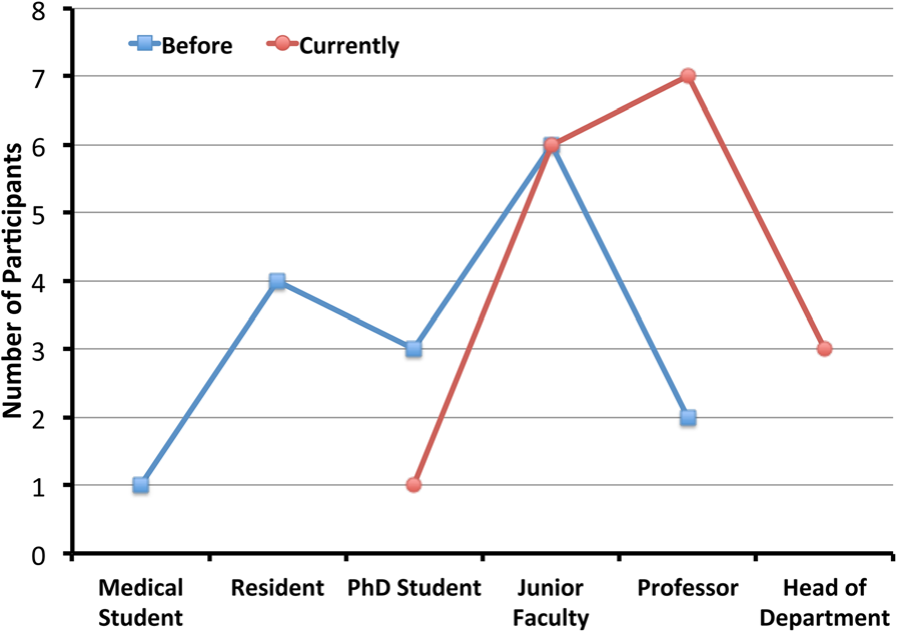
Graphical description of the academic careers of exchange program participants who are currently employed at Kazan State Medical University (*n* = 15). Note the rightward shift in academic positions of current KSMU employees

The exchange program enhanced career development of participants employed at KSMU. Figure 5 illustrates the shift in academic positions over time among the group of KSMU program participants. Many program alumni now hold senior and key academic positions within the university with the ability to influence curriculum and educational methods. Furthermore, three participants have been promoted to the head of their department, and two hold clinical leadership positions as section chiefs at KSMU-affiliated hospitals.

### Changes to KSMU’s curriculum as a result of the collaboration

The following courses and teaching methodologies introduced by faculty from YSM/WCHN/UVMCOM have since been developed and incorporated into KSMU curriculum by local faculty trained in the U.S.

#### Evidence-Based Medicine

Evidence-based medicine (EBM) concepts were introduced through annual Yale/WCHN/UVMCOM faculty visits to KSMU in the form of lectures given on specific topics, implementation of guidelines, and demonstration through bedside teaching. Later, a case-based EBM curriculum for KSMU was developed and taught by visiting U.S. residents (described in detail by Korthuis et al.[11]). An EBM module curriculum based on the needs of the host institution was designed and translated into Russian. Participants had access to *Evidence*-*based Medicine*: *How to Practice and Teach EBM*, a textbook by Sackett et al.[12], which was donated to KSMU by the Centre for Evidence-Based Medicine (Oxford, United Kingdom). This EBM teaching model was initially used by the Department of Internal Diseases of KSMU for postgraduate education, but was adopted shortly thereafter by other major clinical departments including Adult and Pediatric Infectious Diseases, Obstetrics and Gynecology, and Surgery. Furthermore, several KSMU faculty from the Departments of Internal Diseases and Infectious Diseases continued teaching the EBM course independent of visiting faculty and residents after receiving formal training in this discipline in the U.S. for a minimum of 4 months. Finally, EBM teaching was introduced to undergraduate medical education, first to international and then to Russian students. Presently, all students of KSMU’s General Medicine faculty are taught the principles of evidence-based medicine. Since 2007, 171 International students have completed this course, while 325 KSMU students, residents, and faculty have received certification in basic or advanced EBM from American faculty.

#### HIV/AIDS teaching course

The HIV/AIDS course was one among many courses taught annually by YSM/WCHN/UVMCOM faculty at KSMU. In kind with establishment of the EBM course, several faculty members from the Department of Infectious Disease were trained at Yale/WCHN/UVMCOM through the exchange program with the goal of eventually teaching an HIV/AIDS medicine course independently at KSMU. Initially taught by American faculty and attended primarily by physicians and senior residents, the teaching of this course was later transitioned to KSMU faculty who offered it to a broader audience, including medical students, in Russian. Finally, KSMU integrated this course into the fifth-year medical student curriculum in the Department of Infectious Diseases.^5^ In addition, American faculty published several scientific review articles with Russian translation in a local medical journal to reach a wide audience [13, 14]. Over the past 5 years, 250 faculty members and senior residents were trained via this lecture course. Since inception of the partnership more than 600 faculty and senior residents have received formal training in HIV/AIDS medicine, a course centered on epidemiology, diagnosis, prevention, and treatment of HIV/AIDS. Due to growing demand for HIV/AIDS training, the course has also been offered to physicians in Naberezhnye Chelny, an official distant branch of KSMU as well as to practicing physicians undergoing relicensure and recertification every 5 years as required training by the Federal Ministry of Health.

#### Teaching how to teach course

Based on the Stanford Faculty Development Program Model, which has been extensively proven effective for clinical teaching [15–17], this course was designed to improve the quality of medical teaching and create a conducive clinical learning environment. The instructor was a faculty member with many years of experience teaching this course to faculty and medical residents in the U.S. The course was used as a basis for faculty development workshops, conducted on three different occasions – 2001, 2003, and 2009 – during which 98 KSMU faculty members were trained. The details of the Stanford Faculty Development Program were first published in a Russian-language journal [18] prior to the initial teaching visit in 2001. Results of the implementation of the faculty development course have been published separately [19, 20] and revealed a positive and lasting effect on the teaching skills of KSMU faculty members despite significant differences between the American and Russian cultures of medical education. The multitude of clinical faculty trained through this program ensured that the main principles of clinical instruction provided by the Stanford Model were implemented across most clinical disciplines in KSMU. Furthermore, the evaluation process introduced through the Stanford Model is now routinely used to assess the efficacy of teaching, and serves as the basis for annual faculty evaluations and feedback.

#### Tropical medicine course

Growing demand for a tropical disease course by international students at KSMU lead to its emergence from a general infectious disease course taught annually by YSM/WCHN/UVMCOM faculty. The scarcity of tropical diseases in this part of Russia rendered tropical diseases a small component of the infectious disease curriculum. However, international students training to work in Asia, the Middle East, and former Soviet countries, in which tropical diseases are more prevalent, needed to be competent in the topic. To this end, an infectious disease specialist from KSMU returning to Russia after a 6 month infectious diseases training at WCHN co-authored a 1 week annual curriculum with a WCHN faculty member. Designed based on the needs of KSMU medical students, this course has been translated to Russian language and is open to all students, national or international. Three-hundred students, residents and faculty over the past 5 years, and over 600 since the inception of the partnership, have successfully completed this tropical medicine course.

#### “Clinical teaching team structure”

Having had ample exposure to the concept of “Clinical Teaching Team Structure,” KSMU’s Pediatric Infectious Diseases Department was the first to integrate patient care into student and resident medical education. The department head, as well as many faculty members, had visited and/or trained at Yale/WCHN/UVMCOM, while many faculty and residents from these institutions had frequently visited KSMU.

Adoption of the “Clinical Teaching Team Structure” was initially challenging because it was not customary in the Russian medical education system for interns, residents, and students to study together. However, special efforts were made to assemble teams composed of one faculty member, two residents, and two to three medical students per infectious disease rotation. This system positively impacted patient care and education of residents and students. It also provided medical students with more hands-on practice and patient engagement while reducing classroom time. The Department of Internal Diseases recently introduced the “Clinical Teaching Team Structure,” first as a pilot project and recently on a permanent basis. The positive experiences of these two departments incited interest in other KSMU departments to adopt this model of patient care and clinical education.

#### Introduction of a global health elective

A 6 week global health training elective program was established in Uganda for Russian physicians to educate KSMU physicians and medical trainees about the practice of clinical medicine and the general practice of global health in culturally diverse, resource-limited settings. The project was a multi-institutional effort amongst KSMU, YSM, WCHN/UVMCOM, and Makerere University in Kampala, Uganda. Over the past 5 years, the training of 20 KSMU affiliates in Uganda has demonstrated the program’s efficacy (published separately [21]).

## Discussion

This study describes a successful international cross-cultural collaboration between one Russian and two American universities over 20 years with the goal of modernizing and improving the Russian medical education system via capacity building. This has been a unique partnership in medical education as longevity is universally difficult for international partnerships to attain. Lack of mutual respect and reciprocity, unidirectionality, incongruent objectives, cultural clashes, changing priorities of collaborating institutions, and funding limitations stand as ubiquitous obstacles. Several factors serving as the structural pillars have upheld the structure of the collaboration beneath the surface, superseding these obstacles and ultimately leading to its success. The passion, persistence, and devotion of key involved individuals to the program prompted them to do the legwork on their own, hosting guests for months without financial incentives while the program was growing. Embedded in every cross-institutional interaction were mutual respect and trust, complete transparency and accountability. The program was lead by ideals that were visionary and risky, but also practical and flexible. Furthermore, establishment of this program was auspicious given the difficult situation of the Russian healthcare system at the time of its inception [1–3]. To the best of our knowledge, no other long-term, successful collaborations have subsisted amongst any regional Russian medical universities and American medical institutions.

Perhaps the most significant change transpired in KSMU has been the culture of evidence-based medicine and, transitively, the challenging of authority and opening of dialogue between students and teachers. Originally, decisions regarding the best diagnostic or treatment strategy in the hospital were based on the experience of the most authoritative or senior member, or as dictated by central government officials [22], rather than on evidence-based medicine. Authority was not to be challenged. The implementation of evidence-based medicine was thus a difficult feat for both individuals and institutions [22] as the concept questioned a long-standing culture of authority rule. Comfort with the concept was facilitated by the teaching of evidence-based medical strategies by knowledgeable and respected YSM/WCHN/UVMCOM clinical faculty along with the trust from senior members first that their medical system was in need of improvement, and second that these concepts could help achieve that end. This integration of evidence-based medical curriculum, along with other courses, was additionally challenging because the curricula of higher medical educational institutions in the Russian Federation are determined by the Federal Ministry of Health Care. Universities were not permitted to modify the curriculum per local needs and traditions beyond a trivial extent [23].

Despite the many obstacles, these concepts and methods subsisted beyond their introduction thanks to the many physicians and program alumni trained in these principles who are now senior members of their respective departments and hold faculty positions at KSMU with the authority to implement their acquired skills and knowledge. This uniquely experienced group of faculty members is an invaluable resource that may foster new national and international collaborations, thereby further enhancing the quality of medical education. In 2003, largely with the help of faculty trained in the U.S., KSMU was able to launch a new English language curriculum for teaching general medicine to international students.

Over the past decade, the quality of medical education at KSMU has significantly improved such that it was ranked as the 3^rd^ best higher medical institution in Russia in the 2014 Academic Ranking of World Universities-European Standard (ARES-2014), after Moscow State Medical University and Saint-Petersburg Medical University [24]. As one of the most competitive medical universities in Russia, KSMU attracts highly competitive students both nationwide and internationally. In fact, the number of international students at KSMU has tripled over the past 5 years.

The cultural changes at KSMU were implemented through stages, at first only subtly by planting seeds of thought. The program challenged the culture of adherence to authority through role modeling and treating learners with respect. It encouraged teachers to be forthcoming when they did not have answers to questions, while encouraging students to find answers themselves to share in the following class. Bedside mannerism was taught through demonstration during clinical rounds. New and advanced methods in medical education were introduced verbally in lectures and discussions and further enforced through demonstration in seminars and at the bedside. Once English language proficiency was recognized as essential for implementing educational changes, a faculty member from the English language department was trained to teach English to interested medical students, English language clubs were initiated across the campus, and an English medical library was established. Libraries serve a critical role in sharing valuable resources, preserving and organizing artifacts and concepts, and bringing together people and ideas. They are a platform upon which ideas can be learned, pondered, expanded, and shared. Thus, they are the pinnacle for any educational institution. This building became a central breeding ground for new ideas and their implementation in the culture of KSMU. In this manner, a culture of English language learning spawned at KSMU.

As these seeds began to sprout and the concepts of medical education reform circulated around the university, we began working on training young faculty at KSMU and inviting junior faculty to Yale/WCHN/UVMCOM, as there was a demand for new teachings. All courses that eventually became integral to KSMU’s curriculum began as a series of lectures for selected interested students or residents in English with simultaneous Russian translation. Topics were then compiled into a syllabus, and the courses taught in conjunction with one or two Russian colleagues. The syllabi were subsequently translated into Russian and used by KSMU faculty to teach the course, at which point each became an integral component of university curriculum. Participants were always given a certificate upon completion of a course to recognize their interest and accomplishment, and to encourage and empower them to teach the course independently at their respective centers. Many participants of HIV/AIDS medicine came from multiple clinical sites outside KSMU.

The collaborative program managed to address some of the most pressing healthcare concerns in Russia at the time, such as the well-documented HIV/AIDS epidemic [25]. By bringing to KSMU the most up-to-date information regarding the natural course of the disease, its diagnosis, and treatment strategies, American faculty members were able to train a significant number of physicians in HIV/AIDS management and to raise awareness about this disease among the medical professionals of Kazan and neighboring cities. The efficacy of such CME-style training programs for physicians in Russia has been shown by The Eurasian Medical Education Program [1].

Several lessons have been learned through the process of constructing this program. Although vast, cultural barriers in the practice of medicine between the U.S. and Russia did not preclude fruitful collaboration. Perhaps this important lesson can be extended to collaborations among other countries. Cultural changes, however, did not occur rapidly but rather over a long period of time. Capacity building is an arduous undertaking that requires time and cannot be rushed. To facilitate the program’s goal of capacity building within country, participants should be carefully pre-selected. One of the unexpected inadequacies of the program was that roughly one-third of participants (primarily students and residents at the time of U.S. training) decided to continue their careers abroad despite the program’s intention of building capacity locally. Although it is not possible to know whether all these individuals would have left Russia had they not participated in the exchange program, the international experience may have influenced that decision. Given this experience, the program shifted its focus on the training of junior faculty who had strong connection with the educational institutions in Kazan. This experience became a hallmark of a similar program developed with Makerere College of Health Sciences [26].

To further extend capacity building, the power of program alumni as a core group of faculty capable of initiating gradual change in undergraduate and graduate medical education should be recognized. Most importantly, this collaboration demonstrated that with persistence, dedication, passion, transparency, and mutual honesty and respect, a true collaboration can supersede cultural, social, or practical barriers.

Over the 20 years of collaboration, three members of the Yale/WCHN/UVMCOM faculty most involved in establishing and maintaining the collaboration as well as teaching at KSMU were honored by the University by being named Honorary (Emeritus) Professors of Kazan State Medical University.

This study has inherent limitations. It is difficult to determine whether the positive changes within KSMU occurred as a direct consequence of the collaborative program, or as a natural evolution in medical education. The higher quality of medical education at KSMU in comparison to many other regional medical universities in Russia suggests that the program facilitated this evolution. Because Russian program participants were selected for training in the U.S. largely based on merit, their higher levels of achievement might have been expected irrespective of their participation in the collaboration. However, this should not preclude interpretation of the study results, because career advancement was not its objective. The study did not have an appropriate control group consisting of KSMU medical students and faculty without any exposure to U.S.-led training programs. Because the collaborative program had a wide influence throughout the institution, it would not have been possible to find a control group within KSMU, and an appropriate comparison group could only be from an outside institution similar in size and scope to KSMU, which would have been difficult to procure for the study. Finally, the retrospective nature of the study provides ground for potential bias in interpretation of results, a study limitation that could not be controlled for.

## Conclusions

Capacity building through the Yale/WCHN-KSMU exchange program has greatly contributed to both the quality of medical education at KSMU and the overall healthcare system of the Republic of Tatarstan. Program alumni have been recognized as a powerful core group of faculty capable of initiating gradual change in undergraduate and medical education. Most importantly, this collaboration demonstrated that with persistence, dedication, passion, transparency, and mutual honesty and respect, a true collaboration beneficial to all parties involved can supersede cultural, social, and practical barriers, and truly thrive.

## Additional files

Additional file 1: Brief description of the Russian medical education model. (PDF 69 kb)
Additional file 2: Yale/WCHN-KSMU exchange program participants’ questionnaire. (PDF 108 kb)
Additional file 3: Questionnaire used to survey the Yale/WCHN-KSMU exchange program participants who are currently faculty of Kazan State Medical University. (PDF 110 kb)
Additional file 4: Additional survey results of exchange program participants who are currently employed at Kazan State Medical University (*n* = 15) illustrating the past and current attitudes towards various aspects of clinical education. Scaling system: 1 – not important at all; 5 – very important. (TIF 1847 kb)

## Abbreviations

AIDS: Acquired immunodeficiency syndrome
CME: Continuous medical education
EBM: Evidence-based medicine
HIV: Human immunodeficiency virus
KSMU: Kazan State Medical University
USAID: United States Agency for International Development
UVMCOM: University of Vermont Robert Larner, M.D. College of Medicine
WCHN: Western Connecticut Health Network
YSM: Yale University School of Medicine

## Footnotes

1
*In Russian postgraduate medical education*, *a “clinical PhD student” is an analogue of a Fellow in the U.S.-based system of medical education.*

2
*Item # 4 of the selection criteria was introduced only after the first year of program’s existence.*

3
*More than one response to question permitted*

4
*Kazan State Medical Academy is also a higher medical education institution that provides postgraduate (not undergraduate) education, including internship and residency training, as well as re-licensure and re-training programs.*

5
*Medical education in the Russian Federation is a 6-year-long program.*
