# Supplementary material for: Developing medical education capacity in Russia: twenty years of experience
Source: BMC Med Educ. 2017 Jan 25;17:24. doi: 10.1186/s12909-017-0861-z (PMC5267488; doi:10.1186/s12909-017-0861-z)
Supplement: Additional file 1: — Brief description of the Russian medical education model. (PDF 69 kb) [file 12909_2017_861_MOESM1_ESM.pdf]

Additional file 1

Medical Education in Russia

In Russia, students are enrolled in medical schools immediately after high school at age 17-18. At the time of enrollment, prospective students need to choose whether they are willing to practice adult or pediatric medicine, as the two are taught separately at two separate faculties. The duration of study is six years. The first two years are fully devoted to studying fundamental sciences (anatomy, physiology, biochemistry, microbiology, etc.), the third year is a transition toward clinical medicine (propaedeutics of internal diseases, pharmacology, basics of general surgery, pathology, etc.), and the final three years are fully clinical and dedicated to three main medical disciplines (medicine, surgery, obstetrics and gynecology) and various sub-specialties. Upon completion of six years of medical school, students are required to take the Final State Examinations consisting of several steps including written knowledge testing, practical skills, patient encounter, and interview with the examination board. Students who successfully pass these examinations are granted a Diploma of a Medical Doctor.

Postgraduate education consists primarily of one or two-year-long training programs. The one-year program is traditionally called an internship, *internatura* in Russian, and primarily trains physicians in general disciplines such as internal medicine, general surgery, etc. although occasionally training in more specialized disciplines is also possible. The two-year program is an analogue of the U.S. residency, *ordinatura* in Russian, and is most commonly used to train in broad disciplines as well as subspecialties. Only upon completion of this program do the MD graduates receive a license to practice medicine in Russia. Some students opt to enter the two-year residency program directly after medical school, while others first complete an internship and then enter a residency program (often a requirement for some subspecialties). The third type

of postgraduate training, *aspirantura* in Russian, is a three-year program designed for students to carry out supervised scientific investigations and results in thesis preparation and defense with a subsequent doctoral degree diploma. This is closest to the U.S. PhD program. In Russia, the three-year program can either be in basic sciences (students can enter this type of program directly after medical school), or in clinical disciplines (available only to individuals having completed the two-year residency program). Currently, the system of postgraduate medical education is undergoing significant changes with the one- and two-year programs being reorganized based on the competency-based-learning approach.

The system of medical education in Russia is described in detail in a recent article by Jargin et al. [1A] which also illustrates the strengths and weaknesses of this educational system.

Reference

1A. Jargin SV. Some Aspects of Medical Education in Russia. American Journal of Medicine Studies 2013;1:4-7.
