# Supplementary material for: Developing medical education capacity in Russia: twenty years of experience
Source: BMC Med Educ. 2017 Jan 25;17:24. doi: 10.1186/s12909-017-0861-z (PMC5267488; doi:10.1186/s12909-017-0861-z)
Supplement: Additional file 2: — Yale/WCHN-KSMU exchange program participants’ questionnaire. (PDF 108 kb) [file 12909_2017_861_MOESM2_ESM.pdf]

Additional file 2

ACADEMIC EXCHANGE PROGRAM YALE-KSMU
Participant questionnaire

Please take a few minutes of your time to fill out this questionnaire. This exchange program is of great value and significance for both Institutions. We hope to receive very sincere feedback from you regarding this project, which will help in its development and improvement in the future.

I. Personal Data

Full Name____

Date of Birth: __/__/____

II. Participation in the Program

Please specify the dates that you participated in the exchange program (month, year):

From __/____ to __/_____

In which specialty (department) did you have the rotation:_____________________

III. Position at the time of participation in the exchange program

- ☐ Student ___ year ______________________faculty
- ☐ Resident (year and department)______________________________
- ☐ PhD student (year and department)____________________________
- ☐ Lecturer (department)______________________________________
- ☐ Associate Professor (department)_____________________________
- ☐ Professor (department)_____________________________________
- ☐ Other (explain briefly)_____________________________________

IV. Present employment and position

Institution/Organization____

Position____

Degree (please specify degree award date)_____________________________

Please list your occupational responsibilities:

- ☐ Clinical work
- ☐ Scientific work in the hospital/clinic
- ☐ Scientific work in the lab
- ☐ Combination of clinical and scientific research activities
- ☐ Teacher
- ☐ Administrative position
- ☐ Other (specify briefly)______________________________________

V. Obtaining information about the program and participating in it

How did you find out about this exchange program project?

☐ Official announcement from KSMU or Yale University (web-site, newspaper, etc)
☐ Heard from a colleague
☐ Was informed by the International Department of KSMU
☐ Was informed by American colleagues
☐ By chance

Did you have to go through an official selection procedure in order to participate in this exchange program?

☐ Yes. **Please specify:** testing, interviewing, questionnaire
☐ No

VI. Your expectation regarding the exchange program

What was your pre-departure goal/aim for participating in the program (please select one most appropriate answer)

☐ to improve my clinical knowledge and skills
☐ to conduct a scientific research project
☐ to improve knowledge of English language
☐ to get acquainted with the system of medical education and healthcare in the USA
☐ to explore the country and travel
☐ other, please specify____

VII. Your exchange program experience

Please describe your activities during the exchange program:

I. Observation and following patients

Observation/Participation in operative procedures:

In the operating room ☐

On the wards ☐ In the clinic ☐

Daily rounds with the team ☐

Night shifts in the hospital ☐

II. Participation in the education process

Morning Reports ☐

Noon/Lunch time reports ☐

Grand Rounds ☐

Work with medical students ☐

III. Laboratory / research work ☐

IV. Using the medical literature resources (library, Internet)

Daily ☐

Once a week ☐

Less frequently ☐

To what extent did the program fulfill your expectations? (Please select one best response)

100%	☐	75%	☐
50%	☐	25%	☐
less than 25%	☐		

In case you responded less than 50% to the above question please specify the reason.

Language barrier ☐
Did not learn anything new ☐
Was not able to participate in a scientific project ☐
Was not able to travel and explore the country ☐

Other____
____

E. Changes

Did you find the knowledge/skills that you obtained during your placement program useful for yourself in the future? (please select one response):

Did not influence my carrier ☐
Helped me in carrier-building in KSMU ☐
Helped me find a new job ☐
Made me want to stay in the USA ☐

After your return from the USA did you continue to work in KSMU?

YES ☐ *NO* ☐

If you answered NO to the previous question, how long were you able to work in KSMU upon return?

Less than a year ☐
1-3 years ☐
3-5 years ☐
I am still a faculty/staff member of KSMU ☐

If you left KSMU, what was the reason?

____
____

Did you sign any contract with KSMU regarding further obligatory employment in the University after your expected return from the USA?

YES ☐ *NO* ☐

F. General Impression about the exchange program

Please give your evaluation of the main components of the placement (one response for each component):

Very bad **1** Bad **2** Neutral **3** Satisfactory **4** Excellent **5**

General organization of the exchange program	1 2 3 4 5
The role of the American side	1 2 3 4 5
Participation of the International Department of KSMU	1 2 3 4 5
Food	1 2 3 4 5
Accomodation in Waterbury	1 2 3 4 5
Accomodation in New Haven	1 2 3 4 5
The trip itself	1 2 3 4 5
Financial support	1 2 3 4 5
Availability of the main resources	1 2 3 4 5
Personnel	1 2 3 4 5

Your comments regarding the main components of the exchange program. Your suggestions are highly appreciated.

____
____
____
____

THANK YOU for your cooperation!
