# Supplementary material for: Developing medical education capacity in Russia: twenty years of experience
Source: BMC Med Educ. 2017 Jan 25;17:24. doi: 10.1186/s12909-017-0861-z (PMC5267488; doi:10.1186/s12909-017-0861-z)
Supplement: Additional file 3: — Questionnaire used to survey the Yale/WCHN-KSMU exchange program participants who are currently faculty of Kazan State Medical University. (PDF 110 kb) [file 12909_2017_861_MOESM3_ESM.pdf]

Additional file 3

Yale-KSMU Exchange Program Faculty Survey

Dear Participant!

Welcome to the Kazan State Medical University-Yale University Office of Global Health Faculty Survey. Please take a few moments to complete this survey, which will help us further analyze the long-term results of the Yale-KSMU exchange program that you participated in.

This study aims to assess the impact of medical training at Yale School of Medicine on Russian medical faculty from Kazan State Medical University. We would like to see if clinical training in a different setting makes a difference in the way participants practice medicine, teach medicine, conduct research, or become leaders.

Participation in this study is completely voluntary. You may refuse to participate or agree to participate. You may stop participation at any time.

The benefits from participation in the study include helping the Yale-KSMU collaboration, Kazan State Medical University, Yale School of Medicine, our research team, and others in the field to better understand the impact of this exchange program on Russian faculty development.

Should you have any questions or concerns regarding this survey, the investigators will be willing to answer them at any time. Please feel free to contact any member of the research group with any such questions:

Dr. Majid Sadigh (majid.sadigh@yale.edu)
Professor Ayrat Ziganshin (ayrat.ziganshin@kazansmu.com)
Dr. Liliya Yausheva (liliya.yausheva@kazansmu.com)
Dr. Bulat Ziganshin (bulat.ziganshin@kazansmu.com)

Please provide your name:

- [Field to fill in full name]

By checking the "yes" box below, I confirm that I voluntarily consent to participate in the research study as described above.

- Yes, I understand the above and agree to participate in the survey
- No, I would prefer not to participate (this option ends the survey)

Instructions

Please enter text in the format requested, or select the best answer. Note that it is not possible to go back and edit your answers once you have progressed to the next page. Therefore, please make sure that you are completely satisfied with your answers for the current page before you click "Next".

1. Are you male or female?
   - Male
   - Female

2. How old are you?
   - [Field for Age in year]

Medical Education and Training

Please provide the information requested for each stage of medical training that you have undergone:

1. Higher Medical Educational Institution:
    - Institution
    - City and country
    - Dates of study
    - Area of focus/Faculty

2. Internship
    - Institution
    - City and country
    - Dates of study
    - Area of focus/Department or specialty

3. Residency
    - Institution
    - City and country
    - Dates of study
    - Area of focus/Department or specialty

4. PhD-Studentship
    - Institution
    - City and country
    - Dates of study
    - Area of focus/Department or specialty

Previous International Medical Training (outside of Russia)

1. Have you ever left Russia for the purpose of medical-related training abroad? (not including the time spent at Yale or Yale-affiliated institutions)
    - Yes
    - No

If you answered "Yes" to the previous question, please fill in the following:

1. Medical Training Abroad # 1
    - Institution
    - City and Country
    - Dates of Study
    - Area of Focus

2. Medical Training Abroad # 2
    - Institution
    - City and Country
    - Dates of Study
    - Area of Focus

3. Medical Training Abroad # 3
    - Institution
    - City and Country
    - Dates of Study
    - Area of Focus

4. Medical Training Abroad # 4
    - Institution
    - City and Country
    - Dates of Study
    - Area of Focus

5. Medical Training Abroad # 5
    - Institution
    - City and Country
    - Dates of Study
    - Area of Focus

6. I have participated in more than 5 medical training experiences abroad
    - Yes (only answer this if you have completed all five of the above entries)
    - No

Current Attitudes 1

Please select one best answer for each question below based on your current attitudes, i.e., how you feel now (at this point in time).

Please rate the importance of the following activities from 1 to 5, with 1 being the least important and 5 being the most important.

1. How important do you think it is for an attending to participate in ward rounds?
2. How important do you think it is for an attending to teach medical students?
3. How important do you think it is for an attending to teach interns?
4. How important do you think it is for an attending to teach residents?
5. How important do you think it is for an attending to teach PhD Students?
6. How important do you think it is for patients or their families to participate in the medical decisions affecting their care?
7. How important do you think it is for the attending to spend time alone with patients and their families to answer their questions about their care?
8. How important do you think it is to inform a patient with a terminal disease, such as cancer, of his/her diagnosis?
9. How important do you think it is to obtain patient consent before performing a procedure, such as a pleural tap or lumbar puncture?
10. How important do you think it is to inform patients and/or their families about complications that occurred after a procedure, such as a pneumothorax after a pleural tap or spinal headache after a lumbar puncture?
11. How important do you think it is for an attending to use Evidence-Based Medicine?

Current Attitudes 2

Please select the one most appropriate response to each question as you feel **at this point in time**

12. How many times a week do you think an attending should participate in ward rounds?
    - Once per week or less
    - Two times per week
    - Three times per week
    - Four times per week
    - Five times per week or more

13. How much time do you think an attending should spend teaching at the bedside?
    - Less than 15 mins per week
    - 15-29 mins per week
    - 30-59 mins per week
    - 1-2 hrs per week
    - More than 2 hrs per week

14. How much time do you think an attending should spend giving didactic teaching sessions such as lectures on general medicine topics?
    - Less than 15 mins per week
    - 15-29 mins per week
    - 30-59 mins per week
    - 1-2 hrs per week
    - More than 2 hrs per week

15. How much time do you think an attending should spend giving seminars focused on specific clinical questions raised by patients under the care of your team?
    - Less than 15 mins per week
    - 15-29 mins per week
    - 30-59 mins per week
    - 1-2 hrs per week
    - More than 2 hrs per week

Current Attitudes 3

Please select the one most appropriate response to each question as you feel **at this point in time**

16. How many times a month do you read a peer-reviewed journal?
    - Less than once per month
    - Once per month
    - Two times per month
    - Three times per month
    - More than three times per month

17. How likely do you think it is that you will still be practicing medicine in Russia in 5 years time?
    - Very unlikely
    - Unlikely
    - Unsure
    - Likely
    - Very likely

Training at Yale – exchange program

1. Dates of time that you spent at Yale
    - Month and year you arrived in Connecticut, USA
    - Month and year you departed Connecticut, USA

2. Distribution of Time in Connecticut (please enter the number of weeks spent at each of the following hospitals)
    - Yale-New Haven Hospital
    - Waterbury Hospital
    - West Haven VA Hospital
    - St. Mary's Hospital
    - Other Hospitals

3. What was your academic position or job title before going to Yale?
    - [Text field to input academic position]

4. How would you characterize your mentors at Yale?
    - Very Poor
    - Poor
    - Fair
    - Good
    - Very Good

5. How would you characterize your relationships with your colleagues (i.e., American physicians) at Yale?
    - Very Poor
    - Poor
    - Fair
    - Good
    - Very Good

6. Did you feel welcomed by the medical community and the medical teams at Yale?
    - Never
    - Rarely
    - Sometimes
    - Very Often
    - Always

Training at Yale – Benefits/Challenges

1. Below is a list of potential benefits of participating in medicine at Yale. What were the **<u>top three benefits</u>** that you experienced? Please rank benefits in order of importance (from 1 -3); Select one answer for each of the items below or type in your own response in "Other" option.
    - Learning different teaching styles, such as bedside teaching, conference learning, and patient-centered education
    - Learning how to incorporate evidence-based medicine into education
    - Learning specific content (increasing knowledge in subspecialty area)
    - Learning research methodology
    - Becoming part of a larger international medical community and network
    - Other (please specify)

2. Below is a list of potential challenges of participating in medicine at Yale. What were the **<u>top three challenges</u>** that you experienced? Please rank benefits in order of importance (from 1 -3); select one answer for each of the items below or type in your own response in "Other" option.
    - Policies or rules that created barriers to your experience
    - Not allowed to participate in hands-on procedures on
    - Not allowed to do histories and physical exams
    - No access to the electronic medical records
    - Financial barriers
    - Differences in the culture of medicine
    - Differences in accents and dialects
    - Other (please specify)

Training at Yale – area of focus

1. What was your area of focus during your time at Yale?
    - [Text Field to fill out area of focus]

2. Did you participate in any regional or national meetings during your stay at Yale?
    - No
    - Yes, Please list below
        - [Text Field to list meetings]

3. Are you still academically involved with your Yale mentors or involved in any projects with Yale-affiliated colleagues?
    - No
    - Yes, Please describe the involvement in the field below
        - [Text Field to describe activities with mentors]

Training at Yale – skills assessment

1. Did you gain specific **<u>clinical skills</u>** during the Yale-KSMU exchange program that have been beneficial to you/your employer since your return to Russia?
    - No
    - Yes, Please describe these skills
        - [Text Field to describe the skills]

2. Did you gain specific **<u>teaching skills</u>** in the Yale-KSMU exchange program that have been beneficial to you/your employer since your return to Russia?
    - No
    - Yes, Please describe these skills
        - [Text Field to describe the skills]

3. Did you gain specific **<u>research skills</u>** in the Yale-KSMU exchange program that have been beneficial to you/your employer since your return to Uganda?
    - No
    - Yes, Please describe these skills
        - [Text Field to describe the skills]

Training at Yale – Challenges upon return to Russia

1. Did you gain encounter any **<u>challenges or barriers</u>** upon returning to Russia?
    - No
    - Yes, Please describe these challenges here
        - [Text Field to describe the challenges]

Training at Yale – Impact on Clinical Medicine and Medical Education in Kazan/Republic of Tatarstan

The Yale-KSMU exchange program encompasses many bilateral exchanges, such as medical students, residents and faculty going from KSMU to Yale, as well as medical students, residents and faculty going from Yale to KSMU.

The following questions ask about the impact of the Yale-KSMU exchange program as whole, including all of the above participants.

1. What are the three most important impacts of the Yale-KSMU exchange program on the **care of patients in Hospitals** of Kazan/Republic of Tatarstan? (Please be specific. If none, please write "none".)
   - [Text Field]
   - [Text Field]
   - [Text Field]

2. What are the three most important impacts of Yale-KSMU exchange program on the **training of undergraduate and postgraduate students** of the Kazan State Medical University? (Please be specific. If none, please write "none".)
   - [Text Field]
   - [Text Field]
   - [Text Field]

Current Career

The following set of questions pertain to your current medical career

1. Current academic position or job title
    - [Text Field to insert job title]

2. Clinical Projects: Please list and describe any clinical projects/collaborations you have initiated or are significantly involved in upon return to Russia that relate to the Yale-KSMU exchange program. If none, please write "none".
    - [Text Field to insert clinical project description]

3. Teaching Projects: Please list and describe any teaching project you have initiated or are significantly involved in upon return to Russia that relate to the Yale-KSMU exchange program. If none, please write "none".
    - [Text Field to insert teaching project description]

4. Research Projects: Please list and describe any research projects that you have initiated or are significantly involved in. This may include basic science, clinical research, or operational research. If none, please write "none".
    - [Text Field to insert research project description]

5. Publications/Scientific Presentations: Please list all publications or presentations in which you are listed as an author. (Provide full citations as applicable). This may include journal articles in refereed journals, posters presented at conferences, oral presentations at conferences, or other scientific presentations. If none, please write "none".
    - [Text Field to insert publications]

6. Current Collaborations: Please list all collaborations in which you are currently involved with physicians/scientists from outside Kazan State Medical University. For each collaboration, please provide the name of the primary collaborator, the organization, and the nature and focus of the collaboration. If none, please write "none".
    - [Text Field to insert current collaborations]

7. Teaching: Please list all clinical education responsibilities that you currently have, and your current method of teaching (didactic, conference, EBM, other). If none, please write "none".
    - [Text Field to insert educational responsibilities]

8. Leadership/Administration: Please list all leadership and/or administrative responsibilities that you currently have. If none, please write "none".
    - [Text Field to insert administrative responsibilities]

Past attitudes

The following questions ask you to think back to the time that you completed your Residency training and report on what your attitudes were at that time.

When you completed your Residency training… (Please rate the importance of the following activities from 1 to 5, with 1 being the least important and 5 being the most important.)

1. How important did you think it was for an attending to participate in ward rounds?
2. How important did you think it was for an attending to teach medical students?
3. How important did you think it was for an attending to teach interns?
4. How important did you think it was for an attending to teach residents?
5. How important did you think it was for an attending to teach PhD Students?
6. How important did you think it was for patients or their families to participate in the medical decisions affecting their care?
7. How important did you think it was for the attending to spend time alone with patients and their families to answer their questions about their care?
8. How important did you think it was to inform a patient with a terminal disease, such as cancer, of his/her diagnosis?
9. How important did you think it was to obtain patient consent before performing a procedure, such as a pleural tap or lumbar puncture?
10. How important did you think it was to inform patients and/or their families about complications that occurred after a procedure, such as a pneumothorax after a pleural tap or spinal headache after a lumbar puncture?
11. How important did you think it was for an attending to use Evidence Based Medicine?

Past attitudes
When you completed your Residency training…
For the next set of questions, please continue to think back to the time that you completed your Residency training. Select the one most appropriate response to each question.

12. How many times a week did you think an attending should participate in ward rounds?
    - Once per week or less
    - Two times per week
    - Three times per week
    - Four times per week
    - Five times per week

13. How much time do you think an attending should spend teaching at the bedside?
    - Less than 15 mins per week
    - 15-29 mins per week
    - 30-59 mins per week
    - 1-2 hrs per week
    - More than 2 hrs per week

14. How much time do you think an attending should spend giving didactic teaching sessions such as lectures on general medicine topics?
    - Less than 15 mins per week
    - 15-29 mins per week
    - 30-59 mins per week
    - 1-2 hrs per week
    - More than 2 hrs per week

15. How much time do you think an attending should spend giving seminars focused on specific clinical questions raised by patients under the care of your team?
    - Less than 15 mins per week
    - 15-29 mins per week
    - 30-59 mins per week
    - 1-2 hrs per week
    - More than 2 hrs per week

16. How many times a month do you read a peer-reviewed journal?
    - Less than once per month
    - Once per month
    - Two times per month
    - Three times per month
    - More than three times per month

17. How likely do you think it is that you will still be practicing medicine in Russia in 5 years time?
    - Very unlikely
    - Unlikely
    - Unsure
    - Likely
    - Very likely

Thank you!

This concludes the survey. Thank you for your time and effort in participating. If you have any additional thoughts or comments you would like to add, please do so in the space below. Otherwise, click "Done" to finish the survey.
