# Supplementary figures and images for: Developing medical education capacity in Russia: twenty years of experience
Source: BMC Med Educ. 2017 Jan 25;17:24. doi: 10.1186/s12909-017-0861-z (PMC5267488; doi:10.1186/s12909-017-0861-z)

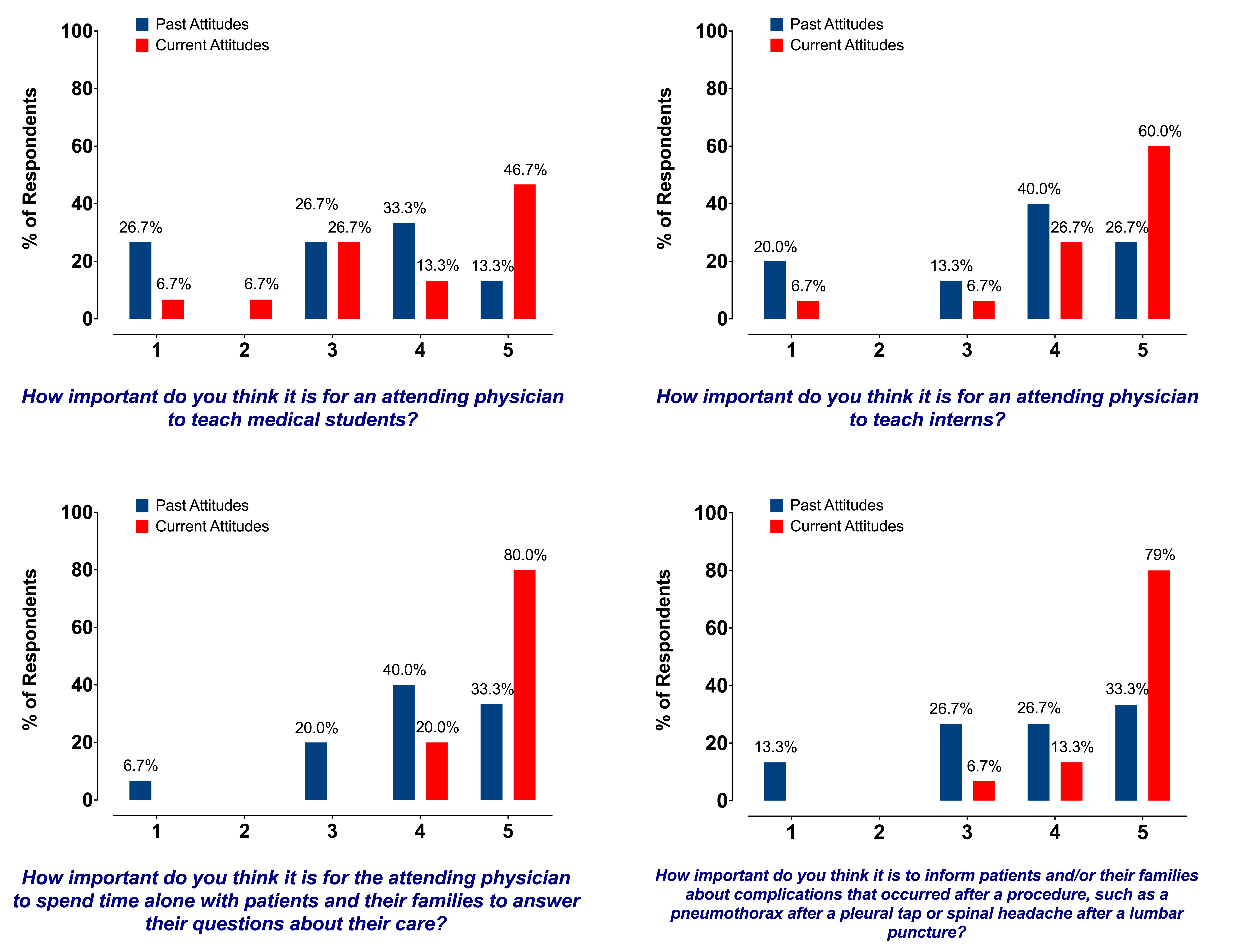

Supplement: Additional file 4: — Additional survey results of exchange program participants who are currently employed at Kazan State Medical University (n = 15) illustrating the past and current attitudes towards various aspects of clinical education. Scaling system: 1 – not important at all; 5 – very important. (TIF 1847 kb) [file 12909_2017_861_MOESM4_ESM.tif]
